# Antagonistic Effects of Lipids Against the Anti-*Escherichia coli* and Anti-*Salmonella* Activity of Thymol and Thymol-β-d-Glucopyranoside in Porcine Gut and Fecal Cultures *In Vitro*

**DOI:** 10.3389/fvets.2021.751266

**Published:** 2021-09-23

**Authors:** Robin C. Anderson, Gizem Levent, Branko T. Petrujkić, Roger B. Harvey, Michael E. Hume, Haiqi He, Kenneth J. Genovese, Ross C. Beier, Toni L. Poole, Tawni L. Crippen, David J. Nisbet

**Affiliations:** ^1^United States Department of Agriculture, Agricultural Research Service, Southern Plains Agricultural Research Center, College Station, TX, United States; ^2^Department of Veterinary Pathobiology, Texas A&M University, College Station, TX, United States; ^3^Department of Nutrition and Botany, Faculty of Veterinary Medicine, University of Belgrade, Belgrade, Serbia

**Keywords:** antibiotic alternative, Salmonella, E. coli, thymol, thymol-β-d-glucopyranoside

## Abstract

Strategies are sought to reduce the carriage and dissemination of zoonotic pathogens and antimicrobial-resistant microbes within food-producing animals and their production environment. Thymol (an essential oil) is a potent bactericide *in vitro* but *in vivo* efficacy has been inconsistent, largely due to its lipophilicity and absorption, which limits its passage and subsequent availability in the distal gastrointestinal tract. Conjugation of thymol to glucose to form thymol-β-d-glucopyranoside can decrease its absorption, but *in vivo* passage of effective concentrations to the lower gut remains suboptimal. Considering that contemporary swine diets often contain 5% or more added fat (to increase caloric density and reduce dustiness), we hypothesized that there may be sufficient residual fat in the distal intestinal tract to sequester free or conjugated thymol, thereby limiting the availability and subsequent effectiveness of this biocide. In support of this hypothesis, the anti-*Salmonella* Typhimurium effects of 6 mM free or conjugated thymol, expressed as log_10_-fold reductions of colony-forming units (CFU) ml^−1^, were diminished 90 and 58%, respectively, following 24-h *in vitro* anaerobic fecal incubation (at 39°C) with 3% added vegetable oil compared to reductions achieved during culture without added oil (6.1 log_10_ CFU ml^−1^). The antagonistic effect of vegetable oil and the bactericidal effect of free and conjugated thymol against *Escherichia coli* K88 tested similarly were diminished 86 and 84%, respectively, compared to reductions achieved in cultures incubated without added vegetable oil (5.7 log_10_ CFU ml^−1^). Inclusion of taurine (8 mg/ml), bile acids (0.6 mg/ml), or emulsifiers such as polyoxyethylene-40 stearate (0.2%), Tween 20, or Tween 80 (each at 1%) in the *in vitro* incubations had little effect on vegetable oil-caused inhibition of free or conjugated thymol. Based on these results, it seems reasonable to suspect that undigested lipid in the distal gut may limit the effectiveness of free or conjugated thymol. Accordingly, additional research is warranted to learn how to overcome obstacles diminishing bactericidal activity of free and conjugated thymol in the lower gastrointestinal tract of food-producing animals.

## Introduction

The gut of pigs can be a reservoir for important foodborne and disease-causing bacteria as well as antimicrobial-resistant populations of pathogenic as well as commensal bacteria potentially selected for by exposure to antibiotics introduced in the production environment. New treatments and strategies are sought to reduce the carriage of these bacteria during rearing and particularly as they are shipped to the abattoir as these bacteria may contaminate the carcass during processing (1, 2). Thymol is an attractive candidate to be developed into an antibiotic alternative because it exhibits potent antimicrobial activity against a variety of bacteria in the laboratory, including zoonotic pathogens such as *Salmonella, Escherichia coli*, and *Campylobacter* (3–6). However, the antimicrobial effect of thymol was less effective when studied in live animals than when studied on the benchtop (7–9) and evidence indicates that the lower activity may be due, at least in part, to intestinal degradation (8) and the rapid absorption of thymol from the proximal gastrointestinal tract (8, 10, 11).

When conjugated to glucose as thymol-β-d-glucopyranoside, the intact glycoside is absorbed to a lesser extent than free thymol in everted porcine jejunal segments allegedly because its β-glycosidic bond is resistant to hydrolysis by host enzymes (10). Consequently, we hypothesized that this compound could potentially function as a prebiotic, being resistant to absorption and degradation in the proximal gut but hydrolyzable by microbial β-glycosidases expressed by gut bacteria inhabiting the distal gut (12, 13). Conceptually, hydrolysis and liberation of thymol from the glycoside in the distal gut would make thymol available to kill zoonotic enteropathogens such as *Salmonella, E. coli*, and *Campylobacter*. In support of this hypothesis, results from *in vitro* studies demonstrated that microbial β-glycosidase activity expressed within mixed populations of avian, bovine, or porcine gut bacteria did indeed hydrolyze thymol from the glucose conjugate, thereby enabling free thymol to exert bactericidal activity against these zoonotic pathogens (14–16). Conversely, when these zoonotic pathogens were grown as pure cultures and in the presence of thymol-β-d-glucopyranoside, little if any bactericidal activity was observed against these zoonotic pathogens due to the absence of appreciable β-glycosidase activity expressed by these bacteria (14–16). Whereas results from *in vivo* studies demonstrated that oral administration of thymol-β-d-glucopyranoside decreased *S. enterica* serovar Typhimurium concentrations in the cecum of weaned pigs and decreased *Campylobacter* colonization in the crop of market aged broilers, bactericidal activity against these pathogens was not observed in more distal sites of the alimentary tract (14, 17). Moreover, both studies revealed little if any effect of thymol-β-d-glucopyranoside on gut concentrations of indigenous *E. coli* (15, 17). Subsequent to the completion of these *in vivo* studies, Van Noten et al. (11) reported that little if any thymol-β-d-glucopyranoside or thymol-α-d-glucopyranoside survived passage through the proximal gut as evidenced by their lack of recovery of detectable amounts of the glycosides in large intestinal contents. Thus, while our findings indicate that thymol-β-d-glucopyranoside can be activated upon reaching microbial β-glycosidase activity expressed by gut bacteria, it appears that this strategy has not yet been optimized to deliver effective concentrations of free or conjugated thymol to the lower gastrointestinal tract. Alternatively, it is possible that the extremely lipophilic character of free thymol and thymol-β-d-glucopyranoside may also contribute to the lack of antimicrobial efficacy observed *in vivo* by favoring sequestration of these compounds within lipid microenvironments during passage through the gastrointestinal tract. Sequestration of free or conjugated thymol would thereby limit accessibility of the compounds to the bacterial cells. Consequently, the objectives of the present study were to assess the antimicrobial activity of thymol-β-d-glucopyranoside within porcine jejunal, cecal, and rectal microbial populations and test the hypothesis that lipids or their hydrolyzed fatty acids may adversely influence the bactericidal activity of thymol-β-d-glucopyranoside as well as its liberated aglycone, thymol, in populations of porcine gut bacteria.

## Materials and Methods

### Sources of Pure and Mixed Microbial Populations

The challenge *S*. Typhimurium strain (NVSL 95-1776), possessing natural resistance to novobiocin, had been made nalidixic acid-resistant *via* successive cultivation in Tryptic Soy broth containing up to 20 μg nalidixic acid ml^−1^ (18). The challenge *E. coli* K88 strain (kindly furnished by Dr. Nancy Cornick, Iowa State University, Ames, IA) was made novobiocin- and nalidixic acid-resistant (NN-resistant) by successive cultivation in Tryptic Soy broth containing up to 25 μg ml^−1^ novobiocin and 20 μg ml^−1^ nalidixic acid (16). Both antibiotics were purchased from Sigma-Aldrich (St. Louis, MO). The *S*. Typhimurium and *E. coli* K88 inocula for experiments were obtained from cultures grown overnight at 37°C in Tryptic Soy broth (Difco, Becton Dickinson, Sparks, MD, USA) supplemented with 25 and 20 μg of novobiocin and nalidixic acid per milliliter, respectively.

Mixed populations of porcine jejunal, cecal, and rectal microbes used as inocula for anaerobic incubations were obtained from respective gut contents freshly collected by necropsy the day of use from a 25-kg weaned pig maintained on a non-medicated diet. Freshly voided porcine feces used similarly for anaerobic incubations were collected from approximately 25 kg conventionally reared weaned pigs maintained on non-medicated feed. All procedures for pig care, euthanasia, and necropsy were approved by the USD/ARS Southern Plains Agricultural Research Center's Animal Care and Use Committee.

### Studies With Jejunal, Cecal, and Rectal Microbial Populations

Freshly collected jejunal, cecal, and rectal contents delivered to the laboratory within 1 h of collection were inoculated (6, 15, and 15 g respectively) into separate 100-ml volumes of anaerobic Mueller Hinton broth (Difco, Becton Dickinson), prepared at half the manufacturer's recommended instruction and under 100% N_2_ gas, to achieve after mixing an approximate fluid turbidity value of 4 on the McFarland standard turbidity scale. Based on reported estimates of approximately 10^11^ total culturable anaerobes g^−1^ of gut contents (19), the inoculum amounts would be equivalent to approximately 6 to 15 × 10^9^ cells ml^−1^ within the jejunal, cecal, and rectal cultures, respectively. The half-strength Mueller Hinton broth was used as the basal medium for the culture of the mixed microbial suspensions to avoid excessive acid production by fermentative microbes. Immediately prior to dispersal, the gut suspensions were inoculated with 10^8^ CFU ml^−1^ of NN-resistant *S*. Typhimurium to achieve an initial count of 10^6^ CFU ml^−1^ within each culture and then distributed (10 ml volumes) to separate triplicate sets of 18 × 150 mm crimp top tubes preloaded with 0.5 ml of water or a 120 mM thymol-β-d-thymol solution to achieve 0 or 6 mM added thymol (Sigma-Aldrich) or thymol-β-d-glucopyranoside (Christof Senn Laboratories, Dielsdorf, Switzerland). Tubes were then sealed and incubated anaerobically under 100% N_2_ at 39°C for 24 h.

### Studies With Fecal Microbial Populations

Freshly voided feces delivered to the laboratory within 1 h of collection were mixed (0.5% wt/vol each) with anaerobic half-strength Mueller Hinton broth to achieve an approximate turbidity value of 3 on the McFarland standard turbidity scale. Fecal suspensions were then inoculated with either the NN-resistant strains of *S*. Typhimurium or *E. coli* K88 to achieve initial concentrations of 10^6^ CFU ml^−1^ of these bacteria. Five- or 10-ml volumes of the resultant mixed populations were distributed under a constant flow of 100% N_2_ gas to 18 × 150 mm crimp top tubes that had been preloaded with small volumes (≤ 0.5 ml) of stock concentrations of thymol-β-d-glucopyranoside or thymol prepared in 50–75% ethanol to achieve 6 mM upon addition of fecal cultures. The smaller culture volumes were used to conserve usage of thymol-β-d-glucopyranoside or thymol and yielded comparable results to the 10-ml cultures. Control tubes without added thymol-β-d-glucopyranoside or thymol were prepared similarly except with additions of 0.5 ml 50% ethanol. In some experiments, tubes were also preloaded with or without 0.15 or 0.3 ml of Crisco Vegetable Oil (J.M. Smucker Company, Orrville, OH, USA), olive oil, oleic acid, or linoleic acid (each from Sigma-Aldrich) to achieve 3% vol/vol solutions as indicated. Similarly, fecal cultures prepared as above were tested without or with additions of Tween 20, Tween 80, or polyoxyethylene-40 stearate (each at 1%, 1%, or 0.2% wt/vol, respectively) or with bile salts or taurine (0.6 or 9 mg ml^−1^ incubation fluid, respectively) to assess the potential impact of emulsions, micelles, or suspensions resulting from inclusion of these additions on pathogen survivability. The surfactants, bile salts, and taurine were purchased from Sigma-Aldrich. Tubes were closed with rubber stoppers, crimped, and incubated anaerobically under 100% N_2_ gas at 39°C for 24 h.

### Bacterial Enumeration and pH Measurements

Viable cell counts of NN-resistant strains of *S*. Typhimurium or *E. coli* K88 were determined in culture fluids collected (1 ml) from *in vitro* incubations after 0, 6, and 24 h on Brilliant Green (Oxoid LTD, Basingstoke, Hampshire, England) or MacConkey (Difco) agars, respectively, with each medium supplemented with 25 and 20 μg novobiocin and nalidixic acid per milliliter, respectively. Samples were serially diluted into 9 ml of phosphate buffered saline (pH 6.5) from 10^−1^ to 10^−6^. Viable counts of wild-type *E. coli* in mixed cultures of jejunal, cecal, and rectal microbes were similarly enumerated on MacConkey agar lacking added antibiotics. Measurements of culture pH were made at the end of each incubation using a pH meter.

### Statistical Analysis

Log_10_ transformations of viable colony counts (CFU ml^−1^) or net changes in log_10_ viable counts of the challenge *S*. Typhimurium and *E. coli* K88 strains, calculated as the difference between counts determined after 6 or 24 h incubation minus counts measured in corresponding cultures at time 0, were tested for main effects of treatment and applicable interactions at each sampling time using a general analysis of variance and LSD separation of means (Statistix10, Tallahassee, FL, USA) with a *p* < 0.05 level of significance. All *in vitro* incubations were conducted with *n* = 3 experimental units per treatment.

## Results and Discussion

**Figure 1:**
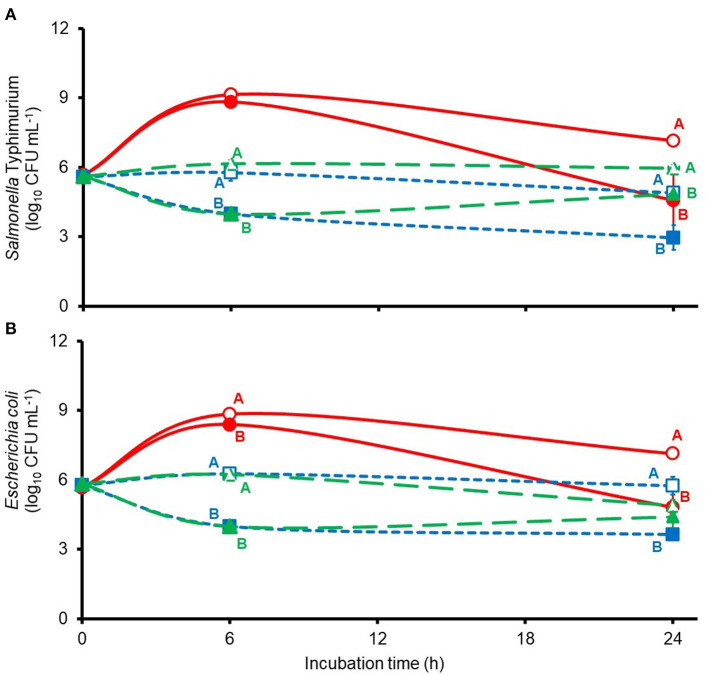
Viable counts of challenge *Salmonella* Typhimurium [graph **(A)**] or wild-type *E. coli* [graph **(B)**] during mixed culture of porcine jejunal (solid red lines), cecal (dashed blue lines), or rectal bacteria (large dashed green lines) treated without (open symbols) or with 6 mM thymol-β-d-glucopyranoside (filled symbols) in anaerobic (under 100% N_2_) ½-strength Muellar Hinton broth. Jejunal, cecal, and rectal means at each sample time accompanied by unlike uppercase red, blue, and green letters, respectively, differ based on least squares separation of means at *p* < 0.05.

Anaerobic *in vitro* incubation of mixed populations of freshly collected porcine jejunal, cecal, and rectal microbes revealed remarkedly similar responses by the challenge *S*. Typhimurium and the wild-type *E. coli* populations to treatment with 6 mM thymol-β-d-glucopyranoside, with significant or trends for significant differences observed between mean viable counts after 6 or 24 h of incubation (Figure 1). Main effects of thymol-β-d-glucopyranoside treatment were observed on mean *S*. Typhimurium log_10_ CFU ml^−1^ after 6 and 24 h of culture of jejunal (*p* = 0.0974 and 0.0104, respectively), cecal (*p* = 0.0001 and 0.0012, respectivly), and rectal (*p* < 0.0001 and 0.0311, respectively) bacteria. Main effects of thymol-β-d-glucopyranoside treatment were observed on mean *E. coli* log_10_ CFU ml^−1^ after 6 and 24 h of culture of jejunal (*p* = 0.001 and 0.0490, respectively), cecal (*p* = 0.0004 and 0.0024, respectivly), and rectal (*p* < 0.0001 and 0.0824, respectively) bacteria. Considering that intact thymol-β-d-glucopyranoside is reported to exert little to no bactericidal activity against *S. Typhimurium* and *E. coli* K88 (15, 16), it is reasonable to conclude that the anti-*Salmonella* and anti-*E. coli* activities observed here reflect the action of free thymol liberated by the action of β-glycosidase released from the indigenous gut microbial populations. The presence of microbial β-glycosidase activity within the gut microbiota was expected as gut microbes inhabiting the niche of polysaccharide digestion contribute to hydrolysis of β-glycosides (12, 13). In the case of the jejunal cultures, the bactericidal activity was quite modest when measured at the 6-h sampling interval but was prominent when measured at the 24-h sampling time (Figure 1). It is possible, however, that *in situ* thymol-β-d-glucopyranoside hydrolysis may occur sooner within the pig jejunum than in the *in vitro* incubations studied here as bacterial concentrations in the pig jejunum would be expected to be approximately 100-fold greater than in the cultures inoculated with jejunal contents in the present experiment (6% wt/vol inoculum). Moreover, van Noten and colleagues (11) reported appreciable hydrolysis (>30%) of both thymol-β-d-glucopyranoside and thymol-α-d-glucopyranoside within the stomach and small intestine of weaned pigs administered 101 and 88 μmol thymol equivalents kg BW^−1^, respectively, over the course of six equally spaced intervals (2 h apart). These researchers recovered low, albeit non-quantifiable, concentrations of thymol in the small intestine and cecum from these treated pigs but were unable to recover detectable amounts of either thymol-α-d-glucopyranoside or thymol-β-d-glucopyranoside in sampled gut contents collected from more distal regions of the gut (11). Their findings suggest rapid hydrolysis and absorption of these compounds in the proximal gut. It is unknown, however, if oral doses of thymol-β-d-glucopyranoside larger than those used by van Noten et al. (11) may allow some portion of the administered glycoside dose to escape hydrolysis during the 1 and 4 h estimated for digesta passage through the pig stomach and small intestine, respectively (20). In the present *in vitro* study, anti-*S*. Typhimurium and anti-*E. coli* activity of thymol-β-d-glucopyranoside was readily apparent by 6 h incubation of cecal and rectal populations, which suggests that the longer retention times of digesta through the large intestine, which is estimated to take up to 44 h (20), may allow ample time for appreciable hydrolysis of any available thymol-β-d-glucopyranoside arriving to this site.

It is known that the lipophilic characteristic of essential oils such as carvacrol and thymol can cause challenges for pharmaceutical applications of these oils due to poor aqueous solubility and the propensity to sequester within lipidic environments (21, 22). Swine diets often contain 1–5% added fat, and in some cases even higher amounts, to increase the caloric density of diets and aid in reducing dustiness during feeding (23). Considering that the apparent digestibility of fat sources is reported to range from 73 to 80% for weaned pigs and from 59 to 73% for growing pigs (24), it is possible that available lipid in the small intestine and, although less abundantly in the lower intestinal tract, may contribute to limiting the bioactivity of thymol and thymol-β-d-glucopyranoside in the gut. As hypothesized, the bactericidal activity of thymol-β-d-glucopyranoside was dramatically lessened in the present study when the fecal cultures were incubated with 3% added vegetable oil (Table 1). For instance, the net changes (log_10_ CFU ml^−1^) in *S*. Typhimurium populations, calculated as the difference between counts determined after 6 or 24 h incubation minus counts measured in corresponding cultures at time 0, were diminished by 29 and 61%, respectively, in thymol-β-d-glucopyranoside-treated cultures incubated with 3% added vegetable oil compared to counts measured in cultures incubated likewise without added oil (Table 1). Similarly, the net changes in *E. coli* K88 were diminished by 73 and 112% after 6 and 24 h incubation, respectively, in thymol-β-d-glucopyranoside-treated cultures incubated with 3% added vegetable oil compared to counts measured in control cultures incubated likewise without added oil (Table 1). Porcine fecal cultures treated with free thymol also had much weakened bactericidal activity when incubated with 3% added vegetable oil, with anti-*S*. Typhimurium and anti-*E. coli* K88 activity (expressed as net changes in log_10_ CFU ml^−1^) in thymol-treated cultures being diminished more (*p* < 0.05) due to the presence of 3% vegetable oil than that of thymol-β-d-glucopyranoside-treated cultures (Table 1). For instance, anti-*S*. Typhimurium and anti-*E. coli* K88 activity due to thymol was diminished 114 and 107% and by 108 and 119% after 6 and 24 h incubation of vegetable oil-supplemented cultures compared to non-oil-supplemented control cultures, respectively (Table 1).

**Table 1:** Effects of added vegetable oil on anti-*Salmonella* Typhimurium and anti-*E. coli* K88 activities of thymol or thymol-β-d-glucopyranoside (6 mM) and on final pH during mixed culture with porcine fecal microbes.

	**Antimicrobial activity (expressed as change in log**_**10**_ **CFU ml**^**−1**^**)**^**a**^ **and final pH [in brackets]**			
**Treatment**	***Salmonella*** **Typhimurium**		***E. coli*** **K88**	
	**After 6 h incubation**	**After 24 h incubation**	**After 6 h incubation**	**After 24 h incubation**
Without added vegetable oil				
No added treatment	0.98B	0.89B [6.76B]	1.62A	1.42A [6.65B]
Thymol	−4.92E	−4.92E [7.66A]	−4.14D	−4.14C [7.40A]
Thymol-β-d-glucopyranoside	−4.35D	−4.92E [5.36D]	−4.31D	−4.31C [5.88D]
With 3% added vegetable oil				
No added treatment	1.58A	1.84A [6.28C]	0.64B	0.57B [6.29C]
Thymol	0.67B	0.33C [7.34A]	0.35B	0.79B [6.63B]
Thymol-β-d-glucopyranoside	−3.07C	−1.68D [6.77B]	−1.15C	0.52B [4.81E]
Treatment × vegetable oil interaction	*p* < 0.0001	*p* < 0.0001 [*p* < 0.0001]	*p* < 0.0001	*p* < 0.0001 [*p* < 0.0001]
Standard error of the mean	0.141	0.179 [0.156]	0.113	0.121 [0.028]

a *Changes in viable counts of the challenge S. Typhimurium and E. coli K88 strains are the difference between counts determined after 6 or 24 h incubation minus counts measured in corresponding cultures at time 0. Challenge S. Typhimurium and E. coli K88 strains were inoculated to achieve initial counts of 10^6^ CFU ml^−1^. Values in brackets refer to pH. Means within columns accompanied by unlike uppercase letters differ based on least squares separation of means at p < 0.05*.

Measurements of pH after 24 h incubation revealed treatment by oil interactions in the fecal cultures inoculated with the challenge *S*. Typhimurium or *E. coli* K88 strains (Table 1). For cultures incubated without added vegetable oil, greater acidification (*p* < 0.05) was observed in cultures treated with thymol-β-d-glucopyranoside than in treated with thymol only or control cultures not treated with either free or conjugated thymol, which indicates hydrolysis and subsequent fermentation of glucose from the β-glycoside. This finding is consistent with the simultaneous release of thymol in the non-oil-containing cultures treated with thymol-β-d-glucopyranoside, thereby contributing anti-*S*. Typhimurium or anti-*E. coli* K88 activity comparable to that observed in similar cultures treated with thymol. For incubations conducted with 3% added vegetable oil, acidification was observed after 24 h incubation of thymol-β-d-glucopyranoside-treated cultures inoculated with *E. coli* K88 but not with similar cultures inoculated with *S*. Typhimurium (Table 1). This suggests that less substrate was available for fermentation in the *S*. Typhimurium-inoculated cultures incubated with 3% added vegetable oil than in similarly incubated *E. coli* K88-inoculated cultures. It is known that long-chain fatty acids hydrolyzed from vegetable oil by microbial lipases can be inhibitory to anaerobic digestion (25), but the reason for the discrepancy in pH between *S*. Typhimurium- and *E. coli* K88-inoculated cultures incubated with 3% added vegetable oil is unclear. It is unlikely that these different pH conditions had an appreciable effect on viability of the challenge *S*. Typhimurium or *E. coli* K88 strains as others have reported growth and survivability of *S*. Typhimurium and *E. coli* were little affected, if at all, by pH ranging from 5 to 7 (18, 26). Kampfer and colleagues (27) reported the hydrolysis of 4-methylumbelliferyl-β-d-glucopyranoside in 16 and 83%, respectively, of 43 *E. coli* and six *Salmonella enteritidis* strains they tested; thus, one possible hypothesis for the differential pH response of mixed populations inoculated with *S*. Typhimurium or *E. coli* K88 is that thymol-β-d-glucopyranoside-hydrolyzing enzymes may have been expressed by *E. coli* K88 but not *S*. Typhimurium used in the present study. In support of this hypothesis, Levent et al. (16) showed that when grown in pure culture, thymol-β-d-glucopyranoside treatment had no antibacterial activity against *S*. Typhimurium but did against *E. coli* K88, thus implicating hydrolysis of thymol from the thymol-β-d-glucopyranoside by *E. coli* K88. Considering, however, that earlier studies demonstrated little if any antimicrobial activity of intact thymol-β-d-glucopyranoside (15, 16), the significant inhibition of anti-*E. coli* K88 and anti-*S*. Typhimurium activity observed in the presence of 3% added vegetable indicates the sequestration of thymol by the added vegetable oil precludes antimicrobial activity of liberated thymol.

In a follow-up study, we observed that olive oil, like vegetable oil, also inhibited the anti-*S*. Typhimurium and anti-*E. coli* K88 activity of thymol and thymol-β-d-glucopyranoside as did the prominent fatty acids of vegetable and olive oil, and linoleic and oleic acid (Table 2). In the case of fecal cultures inoculated with *S*. Typhimurium, the anti-*Salmonella* activity of 6 mM added thymol-β-d-glucopyranoside after 6 h incubation was diminished by 36–60% by oil or fatty acid supplementation when compared to cultures incubated without added oil of fatty acid (Table 2). After 24 h incubation, however, the anti-*Salmonella* activity of 6 mM added thymol-β-d-glucopyranoside was diminished to a greater extent by supplements with the oils (66–87%) than by their respective fatty acids (36–42%), although the differences were not necessarily significant (Table 2). In the case of fecal cultures inoculated with *E. coli* K88, the anti-*E. coli* K88 activity of 6 mM added thymol-β-d-glucopyranoside treatment was diminished by 62–82% after 6 h incubation (Table 2). After 24 h incubation, the anti-*E. coli* K88 activity of 6 mM thymol-β-d-glucopyranoside treatment was diminished by 91–112% with oil addition and was diminished to a lesser extent (45–70%) in cultures incubated with added linoleic or oleic acid (Table 2).

**Table 2:** Effect of different oil substrates or their predominant free fatty acids on anti-*Salmonella* Typhimurium and anti-*E. coli* K88 activities of thymol-β-d-thymol (6 mM) during mixed culture with porcine fecal microbes.

	**Antimicrobial activity (expressed as change in log**_**10**_ **CFU ml**^**−1**^**)**^**a**^			
**Treatment**	***Salmonella*** **Typhimurium**		***E. coli*** **K88**	
	**After 6 h incubation**	**After 24 h incubation**	**After 6 h incubation**	**After 24 h incubation**
No added oil or fatty acid without thymol-β-d-glucopyranoside	1.69A	1.65A	0.23A	0.40AB
No added oil or fatty acid with added thymol-β-d-glucopyranoside	−4.39C	−4.39D	−2.47C	−4.50E
3% Vegetable oil with added thymol-β-d-glucopyranoside	−1.84B	−1.49BC	−0.62AB	−0.37BC
3% Olive oil with added thymol-β-d-glucopyranoside	−1.73B	−0.56B	−0.48AB	0.97A
3% Oleic acid with added thymol-β-d-glucopyranoside	−2.08B	−2.58C	−0.66AB	−1.37C
3% Linoleic acid with added thymol-β-d-glucopyranoside	−2.36B	−2.80CD	−0.93B	−2.47D
Treatment effect	*p* < 0.0001	*p* = 0.0002	*p* = 0.0018	*p* < 0.0001
Standard error of the mean	0.281	0.058	0.322	0.500

a *Changes in viable counts of the challenge S. Typhimurium and E. coli K88 strains are the difference between counts measured in cultures after 6 or 24 h incubation minus counts measured in corresponding cultures at time 0. Challenge S. Typhimurium and E. coli K88 strains were inoculated to achieve initial viable counts of 10^6^ CFU ml^−1^. For comparison, the change in concentrations of S. Typhimurium and E. coli K88 in 6 mM thymol-β-d-glucopyranoside-treated cultures grown in medium lacking added vegetable oil were −4.37 ± 0.08 and −4.53 ± 0.41 log_10_ CFU ml^−1^ after 6 h and were −4.37 ± 0.08 and −4.92 ± 0.03 log_10_ CFU ml^−1^ after 24 h incubation, respectively. Means within columns accompanied by unlike uppercase letters differ based on least squares separation of means at p < 0.05*.

To assess the potential effects of lipid dispersal agents on the inhibitory effect of vegetable oil on anti-*S*. Typhimurium and anti-*E. coli* K88 activity of thymol, added as free thymol or derived *via in situ* hydrolysis of thymol-β-d-glucopyranoside, fecal cultures were incubated with additions of bile salts or taurine (0.6 or 9 mg ml^−1^, respectively). Results revealed that fecal cultures incubated with additions of bile salts or taurine alone with thymol or thymol-β-d-glucopyranoside had no effect (*p* > 0.05) on the growth of the inoculated challenge strains of *S*. Typhimurium or *E. coli* K88 and did not diminish the inhibitory effect of 3% vegetable or olive oil or their prominent fatty acids, linoleic or oleic acid, on these pathogens (not shown). Likewise, co-addition of the commercial emulsifying agents Tween 20, Tween 80, and polyoxyethylene-40 stearate at 1%, 1%, or 0.2% wt/vol, respectively, did not restore anti-*S*. Typhimurium or anti-*E. coli* K88 activity (Table 3). Thus, while addition of bile salts, taurine, or the Tween and polyoxyethylene-40 surfactants to the incubation mixture may have aided in solubilizing the vegetable oil and thymol, whether free or conjugated, the thymol compounds were still confined within lipophilic microenvironments dispersed in the aqueous medium. Other researchers have also reported that Tween 80 has little, if any, benefit in enhancing the antimicrobial activity of thymol (28, 29). This result is likely due, at least in part, to the propensity of the Tweens and likewise polyoxyethylene-40 stearate, to establish oil in water micelles or microemulsions that sequester free or conjugated-thymol away from microbial surfaces. Clearly, further studies are needed to determine if co-feeding thymol-β-d-glucopyranoside with other emulsifying agents may effectively promote anti-bactericidal activity of the thymol moieties in the pig gut.

**Table 3:** Effect of select emulsifiers on vegetable oil-caused antagonism of anti-*Salmonella* Typhimurium and anti-*E. coli* K88 activities of thymol-β-d-thymol (6 mM) and on final pH during mixed culture with porcine fecal microbes supplemented with 6 mM thymol-β-d-glucopyranoside and 3% vegetable oil.

	**Antimicrobial activity (expressed as change in log**_**10**_ **CFU ml**^**−1**^**)**^**a**^ **and final pH [in brackets]**			
**Treatment**	***Salmonella Typhimurium***		***E. coli*** **K88**	
	**After 6 h incubation**	**After 24 h incubation**	**After 6 h incubation**	**After 24 h incubation**
Thymol-β-d-glucopyranoside with no added emulsifier	−1.55B	−0.47C [6.48]	0.62	−1.32B [6.59]
Thymol-β-d-glucopyranoside with 1% Tween 20	0.94A	0.87B [6.70]	0.64	−1.53B [6.64]
Thymol-β-d-glucopyranoside with 1% Tween 80	1.21A	1.81A [6.02]	1.23	0.91A [6.65]
Thymol-β-d-glucopyranoside with 0.2% polyoxyethylene-40 stearate	−1.11B	−1.18D [6.36]	0.28	−1.12B [6.64]
Treatment effect	*p* < 0.0001	*p* < 0.0001 [*p* = 0.3472]	*p* = 0.0905	*p* = 0.0004 [*p* = 0.4560]
Standard error of the mean	0.191	0.172 [0.254]	0.226	0.252 [0.029]

a *Changes in viable counts of the challenge S. Typhimurium and E. coli K88 strains are the difference between counts measured in cultures after 6 or 24 h incubation minus counts measured in corresponding cultures at time 0. Challenge S. Typhimurium and E. coli K88 strains were inoculated to achieve initial viable counts of 10^6^ CFU ml^−1^. For comparison, the change in concentrations of S. Typhimurium and E. coli K88 in 6 mM thymol-β-d-glucopyranoside-treated cultures grown in medium lacking added vegetable oil or emulsifiers were −4.37 ± 0.08 and −4.53 ± 0.41 log_10_ CFU ml^−1^ after 6 h and were −4.37 ± 0.08 and −4.92 ± 0.03 log_10_ CFU ml^−1^ after 24 h incubation, respectively. Values in brackets refer to pH. Means within columns accompanied by unlike uppercase letters differ from untreated means based on least squares separation of means at p < 0.05*.

## Conclusion

Results from the present study confirm previous findings indicating that hydrolyzed thymol-β-d-glucopyranoside and free thymol exhibit potent bactericidal activity against *S*. Typhimurium and *E. coli* K88 when incubated with mixed populations of porcine gut bacteria. As hypothesized, the anti-*S*. Typhimurium and anti-*E. coli* K88 activity of these compounds was decreased in porcine fecal cultures containing 3% vegetable or olive oil or their predominant fatty acids, linoleic or oleic acid. Based on these results, it seems reasonable to suspect that undigested lipid in the distal gut may be one of potentially several factors limiting the *in vivo* effectiveness of free or conjugated thymol, potentially by sequestering the lipophilic thymol components away from microbial cells in the animal gut. Results further show that under the conditions of these tests, the emulsifiers Tween 20, Tween 80, and polyoxyethylene-40 stearate had little, if any, effect in overcoming the lipid-caused inhibition of thymol-β-d-glucopyranoside. Accordingly, additional research is warranted to learn how to overcome obstacles diminishing bactericidal activity of free and conjugated thymol in the lower gastrointestinal tract of food-producing animals.

## Data Availability Statement

The raw data supporting the conclusions of this article will be made available by the authors, without undue reservation.

## Ethics Statement

All procedures for pig care, euthanasia, and necropsy were approved by the USD/ARS Southern Plains Agricultural Research Center's Animal Care and Use Committee.

## Author Contributions

RA, GL, and BP contributed equally to the design and planning of the studies. RA, GL, BP, RH, MH, HH, KG, RB, TP, TC, and DN contributed to the conduct of the study, data analysis, interpretation of results, and writing of the paper. All authors have read and approved the final version of the manuscript.

## Funding

This project was funded in part by National Pork Board Grant 14-077 and by research funds appropriated by the United States Department of Agriculture.

## Conflict of Interest

The authors declare that the research was conducted in the absence of any commercial or financial relationships that could be construed as a potential conflict of interest.

## Publisher's Note

All claims expressed in this article are solely those of the authors and do not necessarily represent those of their affiliated organizations, or those of the publisher, the editors and the reviewers. Any product that may be evaluated in this article, or claim that may be made by its manufacturer, is not guaranteed or endorsed by the publisher.
